# Thoracoscopic intrapericardial lingular segmentectomy for advanced lung cancer following immunotherapy

**DOI:** 10.1111/1759-7714.13938

**Published:** 2021-03-16

**Authors:** Andre Chou, Shah Hwa Chou, Yu‐Wei Liu

**Affiliations:** ^1^ Poznań University of Medical Sciences Poznan Poland; ^2^ Department of Surgery Pingtung Hospital, Ministry of Health and Welfare Pingtung Taiwan; ^3^ Department of Surgery Kaohsiung Medical University Hospital, Kaohsiung Medical University Kaohsiung Taiwan

**Keywords:** immune checkpoint inhibitor, immunotherapy, non‐small cell lung cancer, salvage surgery, thoracoscopic surgery

## Abstract

Very little data exists on salvage surgery in previously unresectable or metastatic disease treated with initial immunotherapy. Only a handful of case reports/series regarding surgery for advanced lung cancer after immunotherapy mention the technical challenges involved. We report the case of a 67‐year‐old female with a left lung squamous cell lung cancer revealed by computed tomography‐guided biopsy. Treatment started with chemotherapy followed by immunotherapy in which a partial response was recorded. Subsequent salvage lingulectomy with the thoracoscopic approach was performed. The patient fully recovered and shows no sign of recurrence at follow‐up 16 months on. Our case discusses the surgical tactics involved in the procedure, highlights similar findings encountered in the literature, and contributes to the few reports therein.

## Graphical abstract

Thoracoscopic intrapericardial lingulectomy following immunotherapy can be safely performed even in the presence of pulmonary hilar fibrosis.

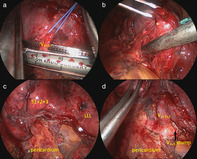

## INTRODUCTION

The use of immune checkpoint inhibitors (ICI) has been gaining ground in the treatment of non‐small cell lung cancer (NSCLC) worldwide. Early studies of these drugs in patients with advanced NSCLC suggest that long‐lasting sound treatment responses in such cases can be expected.^1^ Several studies have evaluated the usage of immunotherapeutic agents as adjuvant or neoadjuvant therapy in surgically resectable NSCLC cases.^2^ However, much is to be learned about the safety and feasibility of lung resection after initial immunotherapy. The literature only has a small number of case reports/series regarding surgery for advanced NSCLC after immunotherapy which allude to the technical challenges entailed.^3^, ^4^, ^5^, ^6^, ^7^, ^8^, ^9^ We herein report one case of advanced lung cancer treated with thoracoscopic lingulectomy after chemo‐immunotherapy.

## CASE REPORT

**FIGURE 1:**
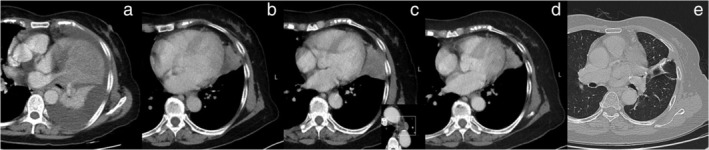
Chest computed tomography (CT) of the patient. (a) Chest CT showed an ill‐defined left lung mass with obstructive atelectasis and massive pleural effusion. (b) Follow‐up CT after platinum‐based doublet chemotherapy (cisplatin plus gemcitabine) for six cycles revealed a notable shrinkage in the left lung tumor (maximum tumor diameter 4 cm). (c) Follow‐up CT revealed evidence of progressive disease for the enlargement of mediastinal lymphadenopathies and the primary tumor (maximum tumor diameter 5.5 cm). (d) Follow‐up CT (just before the surgery) after immunotherapy showed partial response (maximum tumor diameter 3.0 cm). (e) Follow‐up CT 16 months after salvage surgery revealed no evidence of recurrence

A 67‐year‐old woman presented to our hospital with cough and progressive dyspnea. A computed tomography (CT) of the chest revealed an ill‐defined left lung mass with obstructive atelectasis and massive pleural effusion (Figure 1a). Her respiratory symptoms were alleviated after receiving pleural effusion drainage and she subsequently underwent CT‐guided lung mass biopsy for diagnosis. The cytology of pleural effusion revealed metastatic carcinoma and the tumor biopsy revealed squamous cell carcinoma of the lung. Distant metastasis was excluded after bone scan and brain magnetic resonance imaging. The final staging was determined to be cT4N2M1a. She was initiated on chemotherapy (cisplatin plus gemcitabine) for six cycles with partial response (Figure 1b). Three months after chemotherapy, repeated chest CT revealed evidence of progressive disease for the enlargement of mediastinal lymphadenopathies and the primary tumor (Figure 1c). Another four‐cycle platinum‐based regimen (cisplatin plus docetaxel) was delivered. Additionally, the patient was treated with pembrolizumab, a second‐line immune checkpoint inhibitor, for five cycles in total without any complications. The follow‐up CT scan and other restaging work‐ups showed partial response with absence of distant organ metastases (Figure 1d).

**FIGURE 2:**
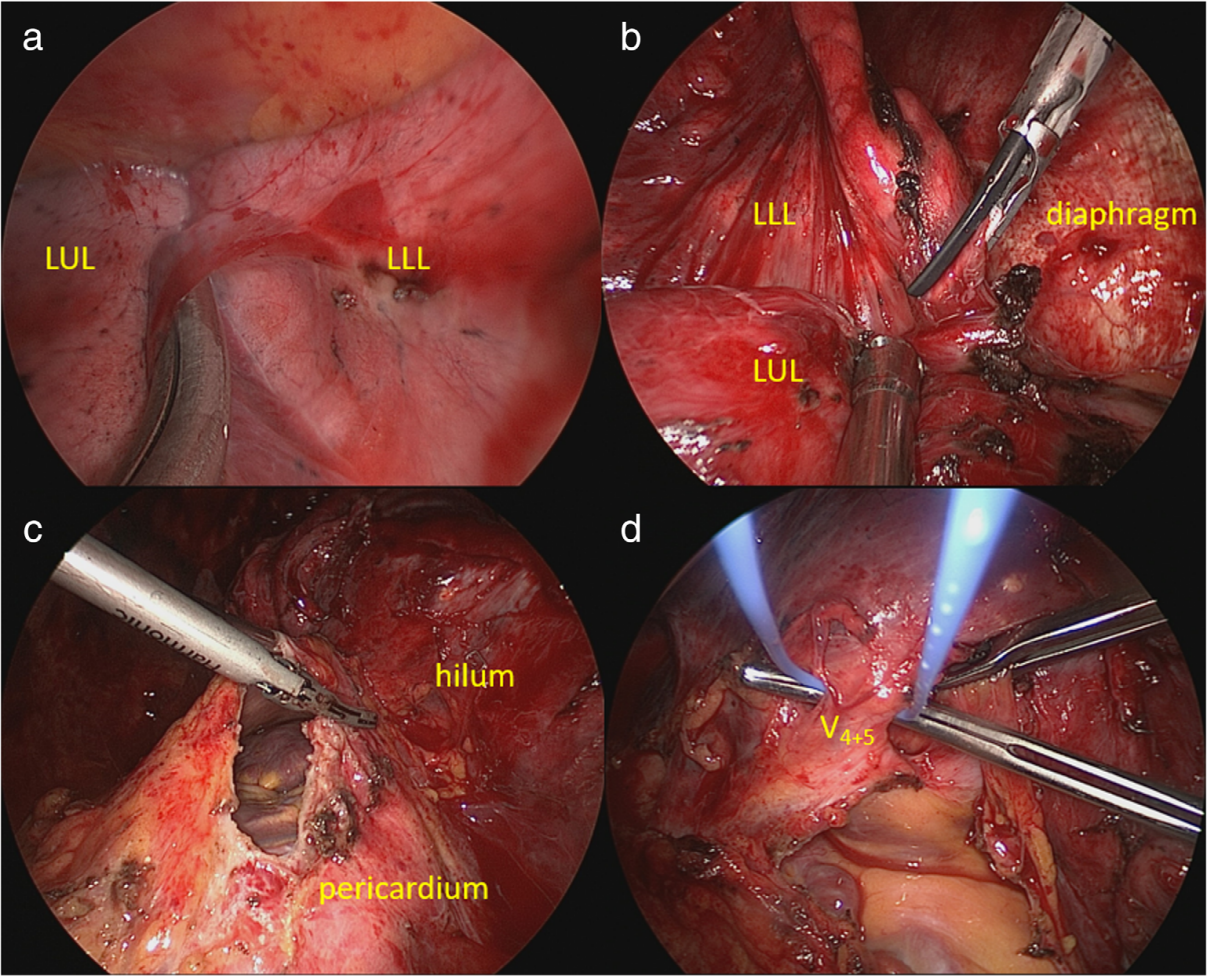
Thoracoscopic view of intrapericardial lingular segmentectomy (part 1). (a) Pneumolysis for diffuse intrapleural adhesions. (b) Dissection of dense adhesions between diaphragm and left lower lung using harmonic scalpel. (c) Opening of the pericardium using electrocautery hook and harmonic scalpel device. (d) Encircle the lingular vein with a vessel loop intrapericardially

**FIGURE 3:**
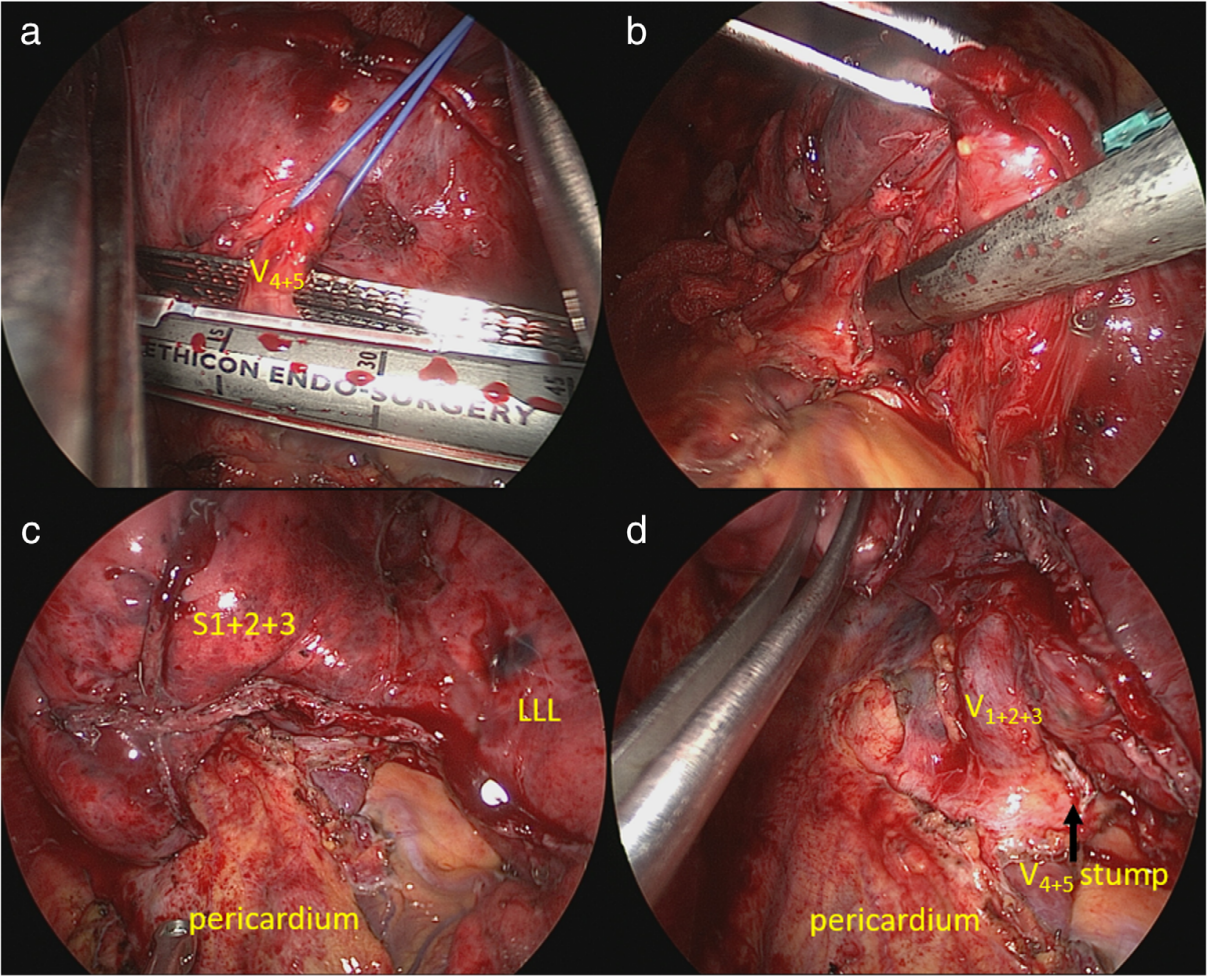
Thoracoscopic view of intrapericardial lingular segmentectomy (part 2). (a) After the lingular vein was encircled and elevated with a vessel loop, dividing with a stapler with a gray cartridge. (b) Using the stapler with a green cartridge for the division of the lingular bronchus and artery and remaining fibrous tissue simultaneously. (c) Intact staple lines along the remaining hilar structure after intrapericardial lingulectomy. (d) The remaining V_1 + 2 + 3_ and V_4 + 5_ stump were observed

Considering the residual tumor after treatment, the patient underwent salvage surgery 4 months after the last cycle of immunotherapy. A double‐lumen endotracheal intubation was performed with right decubitus positioning. The patient underwent left three‐port thoracoscopic approach. Diffuse intrapleural adhesions and dense hilar fibroses were encountered, which were considered remnants of the previous malignant pleural effusion and probably a postimmunotherapy phenomenon (Figure 2(a,b)). In addition to meticulous dissection, the pericardium was opened to avoid directly dissecting fibrotic hilar structures (Figure 2(c)) and enabled an intrapericardial division of the left lingular pulmonary vein with ease (Figure 2(d) and Figure 3(a)). After transecting the lingular vein, we resected the remaining lung using the endostapler together to complete the lingular segmentectomy (Figure 3(b)). Mediastinal lymph node sampling over station 5/6 was also carried out during the procedure. The final pathology showed fibrosis without any evidence of residual malignancy (i.e. pathological complete response). She experienced an uncomplicated hospital recovery and was discharged 3 days postoperatively. To date, the patient has remained disease‐free at follow‐up 16 months later.

## DISCUSSION

The use of immune checkpoint inhibitors (ICI) is gaining traction in the treatment of NSCLC. Hence, a full understanding is imperative due to its potential of becoming a part of mainstay treatment. Several prospective pilot studies revealed the viability of resection after ICI therapy.^3^, ^4^, ^5^, ^6^, ^7^, ^8^ However, perioperative morbidities such as postoperative air leaks and arrhythmia are still frequent occurrences regardless of feasibility and safety.^4^, ^5^, ^6^, ^7^, ^8^ Bott et al. even mentioned more than half of the initially minimally invasive approaches were converted to thoracotomy due to probable hilar inflammation and fibrosis.^6^

Under similar circumstances, we performed intrapericardial division of the lingular vein followed by stapling the remaining hilum due to severe adhesions between the lingular bronchus and artery. In this manner, the danger of pulmonary air leakage and vascular injury can be circumvented. In cases reported by Bott et al. and Fujishita et al., the possibility of a fusion between the pulmonary artery and bronchus following the administration of an ICI has been documented. The former authors cut both artery and bronchus together with a single stapler,^4^ but the latter authors divided the left pulmonary artery and left upper bronchus then cut them individually with staplers after clamping vessels.^4^, ^9^ This technique allowed hemorrhage control in case of vascular injury.^9^

Despite the way immunotherapy has reshaped the treatment approach to NSCLC, dense fibrosis and adhesions of pulmonary hilum may be anticipated during operations based on previous surgical experiences. However, more evidence is still required to validate the data through ongoing clinical trials. From our viewpoint, the merits of intrapericardial division of pulmonary vessels to avoid directly managing/dissecting hilar structure can be considered. Furthermore, thoracoscopic segmentectomy or even lobectomy can be performed safely with careful manipulation using proper surgical techniques.

## DISCLOSURE

The authors report no conflict of interest.

## CONFLICT OF INTEREST AND SOURCE OF FUNDING

None declared.
